# Supplementary material for: The rise in ocean plastics evidenced from a 60-year time series
Source: Nat Commun. 2019 Apr 16;10:1622. doi: 10.1038/s41467-019-09506-1 (PMC6467903; doi:10.1038/s41467-019-09506-1)
Supplement: Supplementary file 1 — Supplementary Information [file 41467_2019_9506_MOESM1_ESM.pdf]

Supplementary Information: The rise in ocean plastics evidenced from a 60-year time series

Clare Ostle et al.

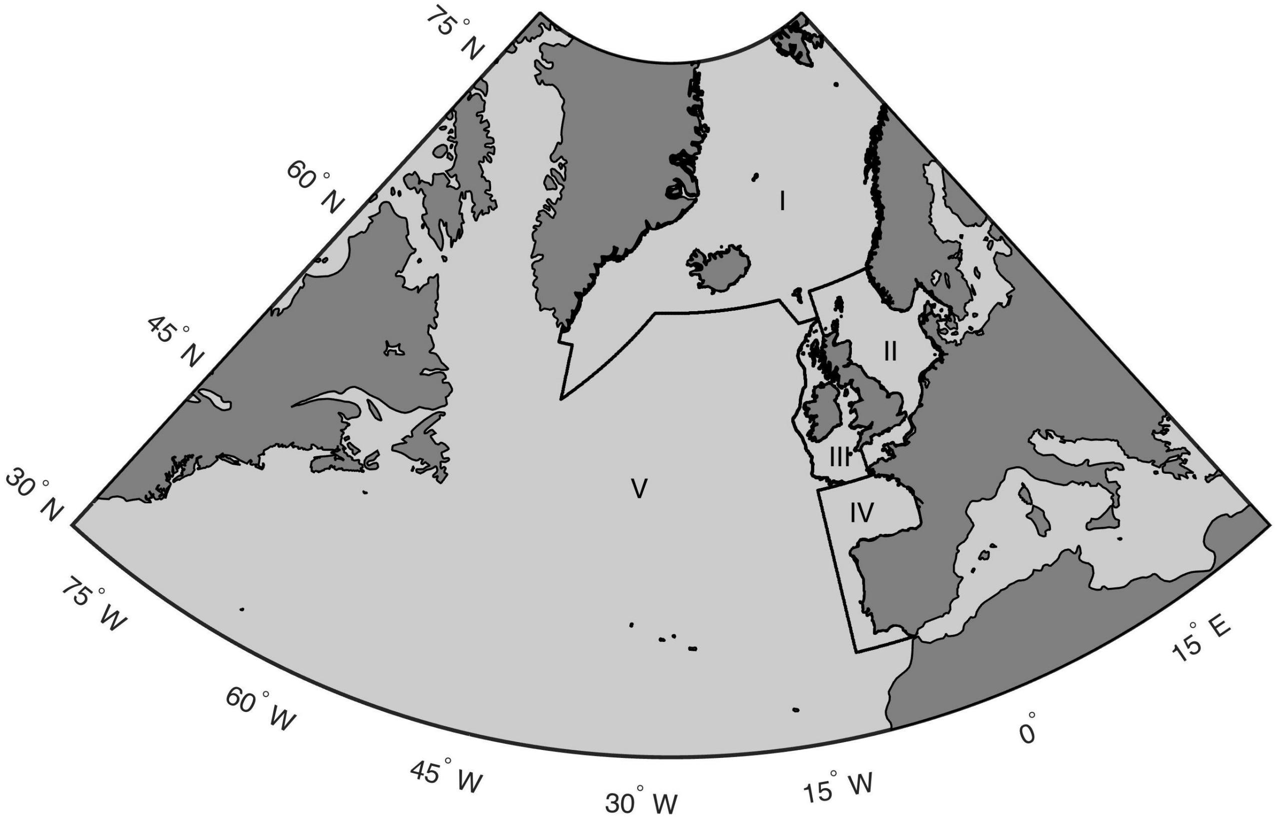

Supplementary Figure 1. Map of regions used. Map of the regions used to divide up the study area, based on OSPAR (Convention for the Protection of the Marine Environment of the North-East Atlantic) regions. I) Arctic Waters, II) Greater North Sea, III) Celtic Seas, IV) Bay of Biscay and Iberian Coast, V) Wider Atlantic. Note: The Wider Atlantic region has been adapted from the OSPAR region to extend to the west of the Atlantic to include the whole study area.

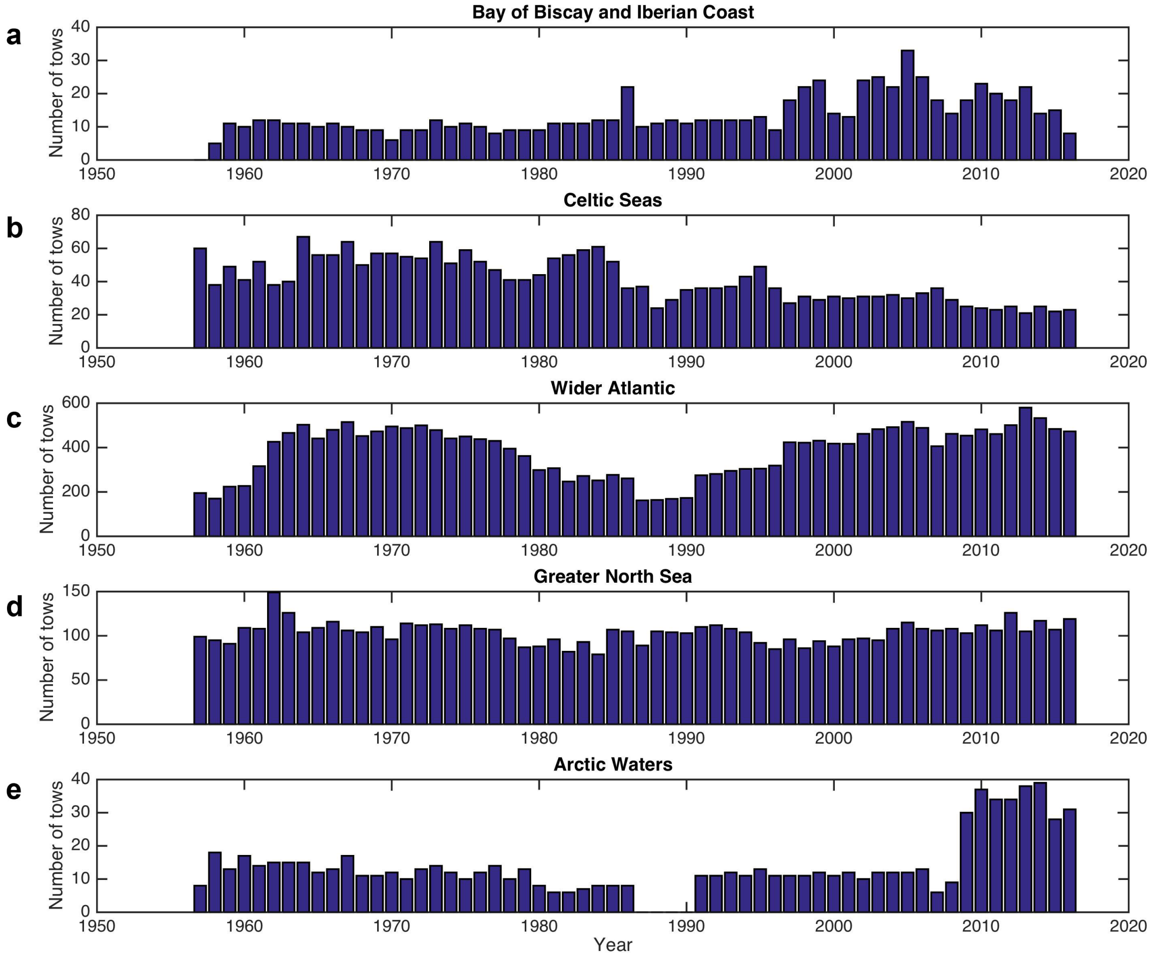

Supplementary Figure 2. Number of tows in each region. Bar chart of the number of Continuous Plankton Recorder tows per year for each region: **a**, Bay of Biscay and Iberian Coast **b**, Celtic Seas **c**, Wider Atlantic **d**, Greater North Sea and **e**, Arctic Waters.

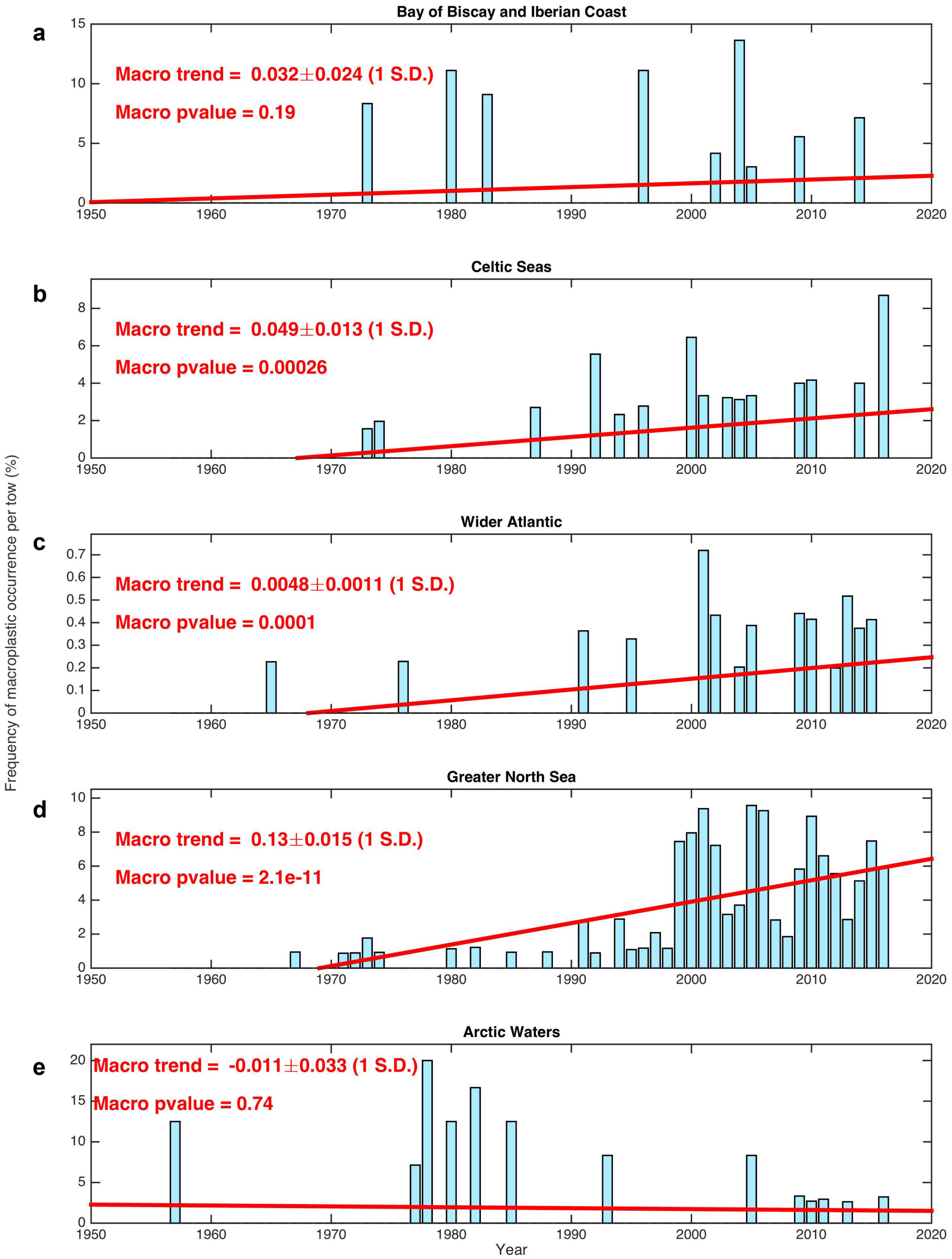

Supplementary Figure 3. Regional macroplastic counts. Annual counts of macroplastic, normalised for number of Continuous Plankton Recorder tows per year = light blue bars, linear trend (standard model I linear regression) shown in red (along with trend and student's t-test pvalue that trend is significantly different from zero) for each region: **a**, Bay of Biscay and Iberian Coast **b**, Celtic Seas **c**, Wider Atlantic **d**, Greater North Sea **e**,and Arctic Waters.

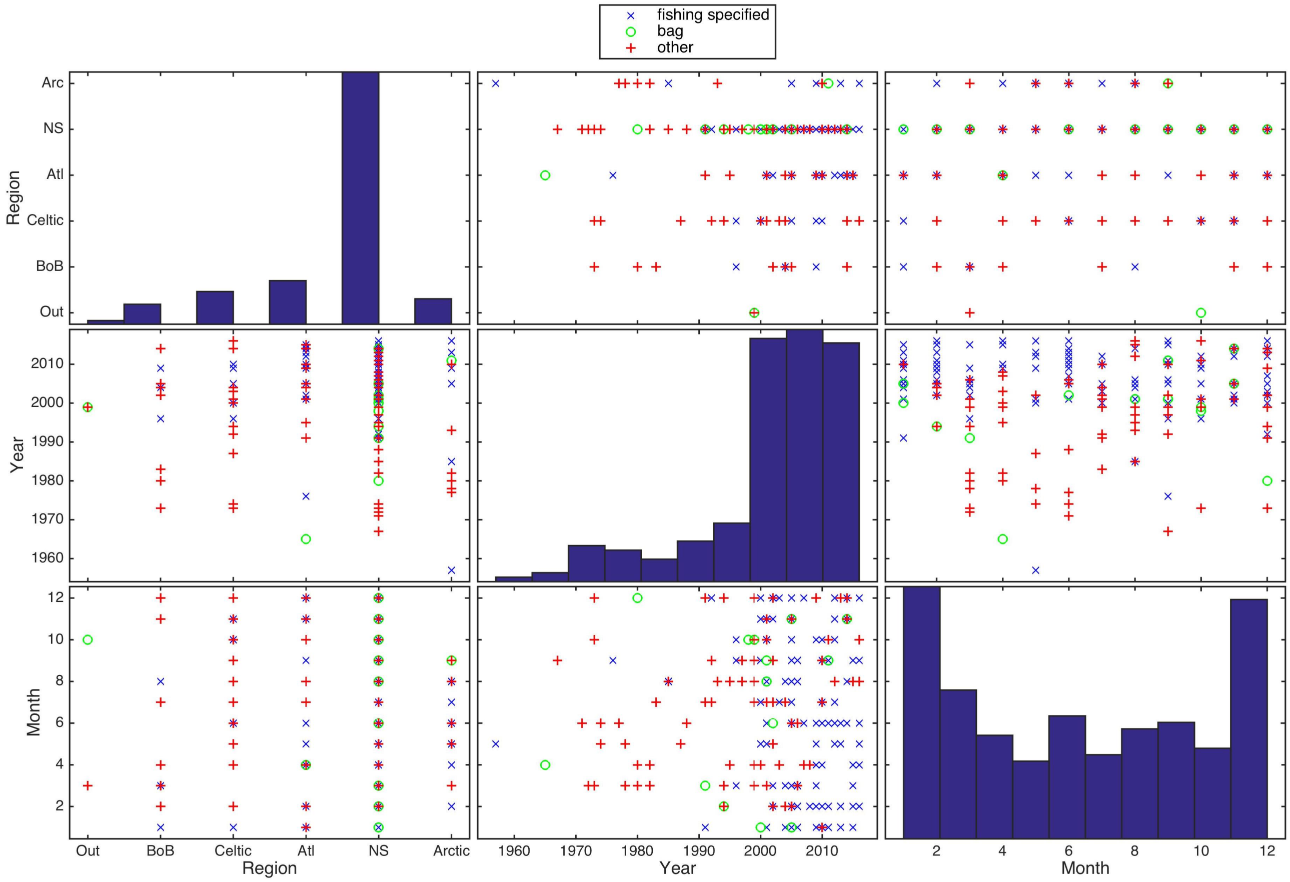

Supplementary Figure 4. Grouped scatter of perMANOVA inputs. Counts of macroplastic divided in to the following marine litter categories: fishing specified (blue ex), bag (green circle), and other (fishing not specified, red cross). Plotted against, year, month and region (Arc=Arctic Waters, NS=Greater North Sea, Atl=Wider Atlantic, Celtic=Celtic Seas, BoB=Bay of Biscay and Iberian Coast, Out=outside of defined regions (Fig. S3)). The diagonal subplots are histograms of the number of macroplastic entanglements per region, year and month respectively.

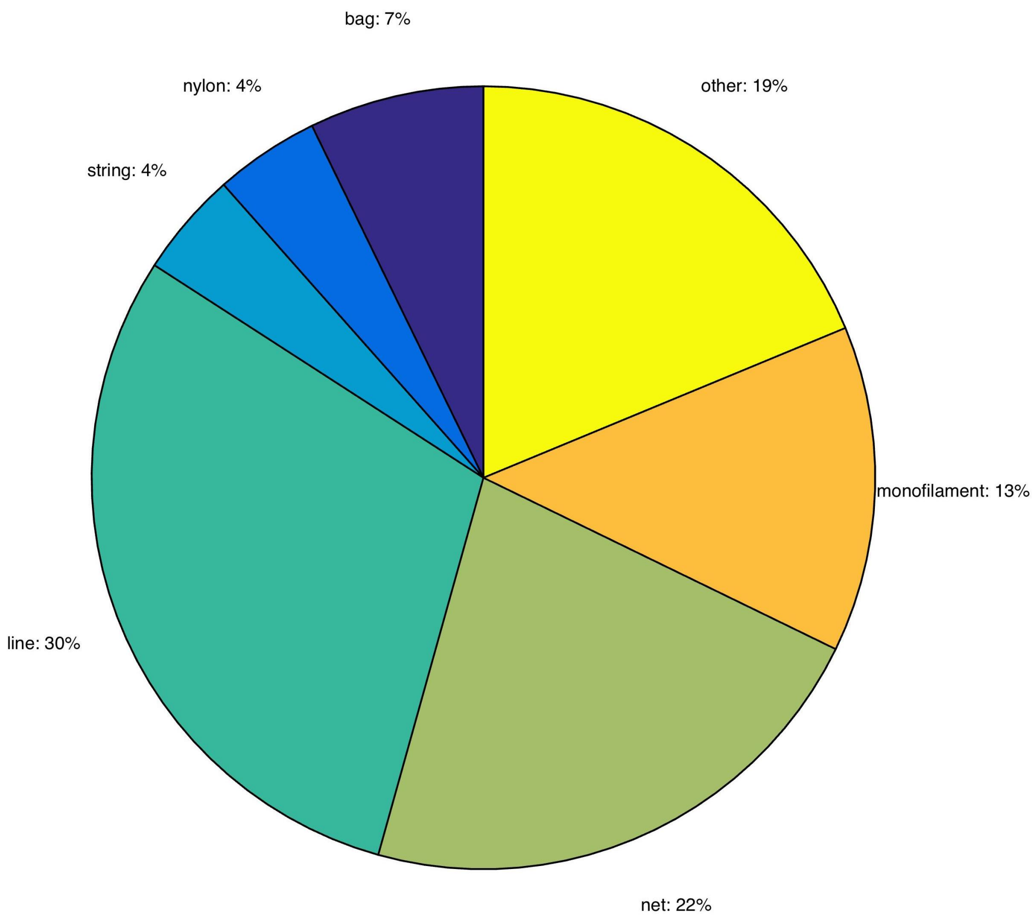

Supplementary Figure 5. Contribution of macroplastic categories. Pie chart of the percentage contribution of different categories to macroplastic counts from the Continuous Plankton Recorder from 1957 to 2016. Note: Those macroplastic records that do not specify a describing word shown in the pie chart are included in the other category.

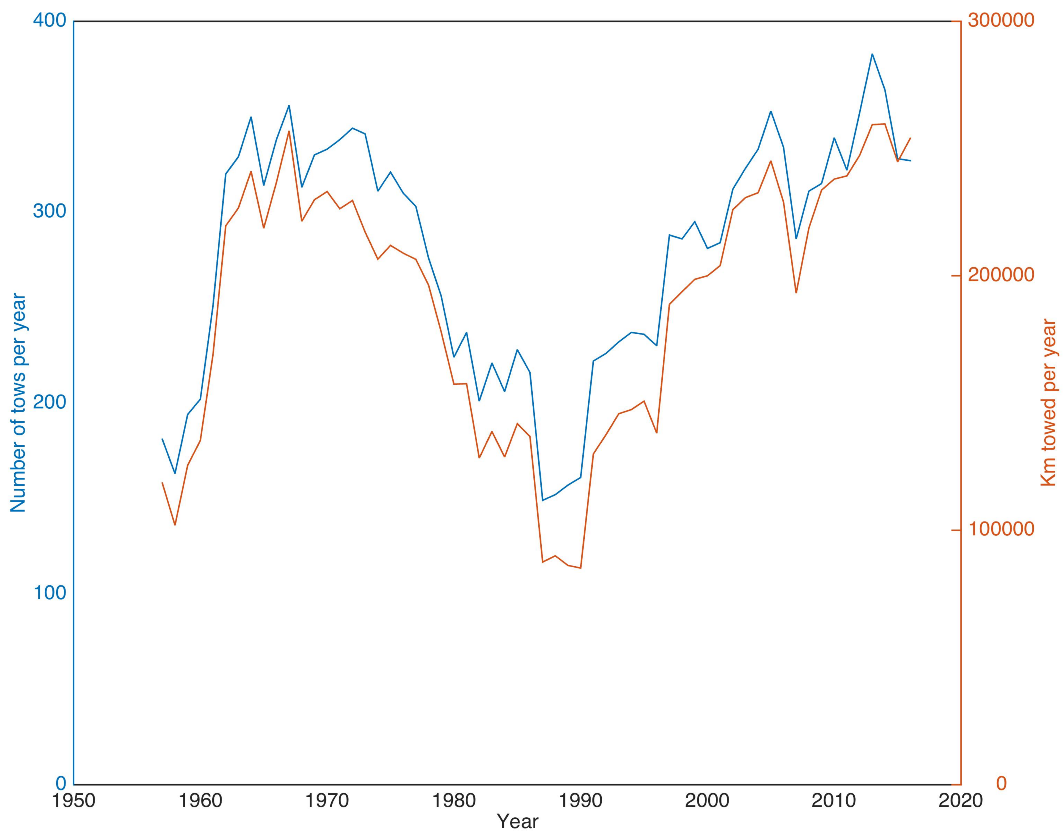

Supplementary Figure 6. Number of km towed and number of tows. Blue = Number of Continuous Plankton Recorder tows per year from 1957 to 2016. Red = Kilometres towed by the Continuous Plankton Recorder per year from 1957 to 2016.

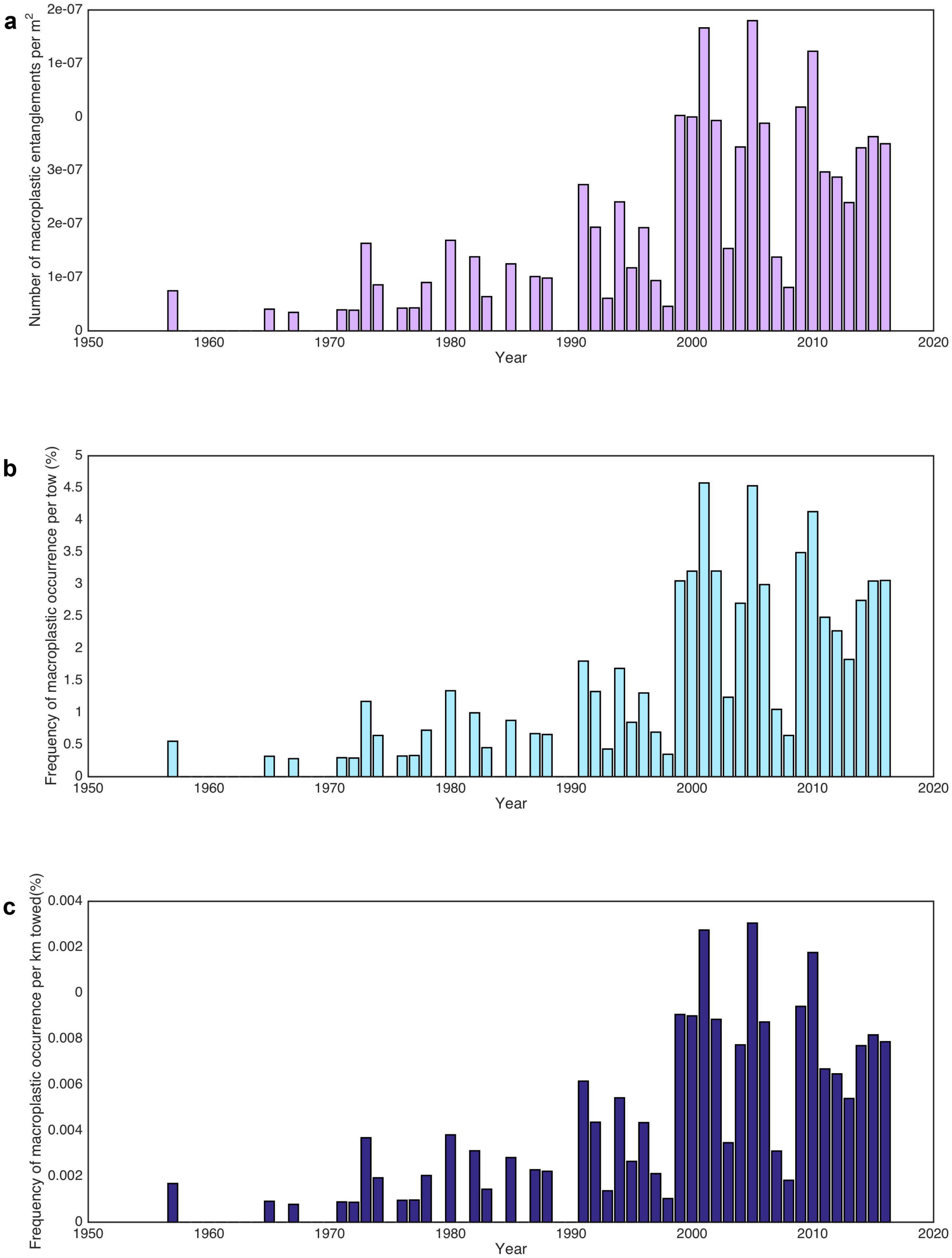

Supplementary Figure 7. Macroplastic entanglements per area covered, tow and per km. **a**, purple bars = number of macroplastic entanglements per area covered (area (m^2) = distance towed (m) x width of CPR (0.225 m)). **b**, light blue bars = frequency of macroplastic occurrence per tow (%). **c**, dark blue bars = frequencey of macroplastic occurence per km towed (%). Note: The normalisation to area and distance towed shown in **a** and **c**, may not take into account sampling bias of the number of tows completed.
