# Supplementary material for: The rise in ocean plastics evidenced from a 60-year time series
Source: Nat Commun. 2019 Apr 16;10:1622. doi: 10.1038/s41467-019-09506-1 (PMC6467903; doi:10.1038/s41467-019-09506-1)
Supplement: Supplementary file 3 — Description of Additional Supplementary Files [file 41467_2019_9506_MOESM3_ESM.pdf]

Description of Additional Supplementary Information

File Name: Supplementary Data 1
Description: Macroplastic entanglements on the Continuous Plankton Recorder.

File Name: Supplementary Data 2
Description: Natural entanglements on the Continuous Plankton Recorder.

File Name: Supplementary Data 3
Description: Annual macroplastic entanglements on the Continuous Plankton Recorder within each OSPAR region (the wider Atlantic region has been adapted from the OSPAR regions to extend to the west of the Atlantic to include the whole study area).
